# Serial progression of cortical and medullary thymic epithelial microenvironments

**DOI:** 10.1002/eji.201344110

**Published:** 2013-12-04

**Authors:** Nuno L Alves, Yousuke Takahama, Izumi Ohigashi, Ana R Ribeiro, Song Baik, Graham Anderson, William E Jenkinson

**Affiliations:** 1 Infection and Immunity Unit, Institute for Molecular and Cellular Biology, University of PortoPorto, Portugal; 2 Division of Experimental Immunology, Institute for Genome Research, University of TokushimaTokushima, Japan; 3 Medical Research Council Centre for Immune Regulation, Institute for Biomedical Research, Medical School, University of BirminghamBirmingham, UK

**Keywords:** Cortex, Medulla, Thymic epithelial cells, Thymus

## Abstract

Thymic epithelial cells (TECs) provide key instructive signals for T-cell differentiation. Thymic cortical (cTECs) and medullary (mTECs) epithelial cells constitute two functionally distinct microenvironments for T-cell development, which derive from a common bipotent TEC progenitor. While seminal studies have partially elucidated events downstream of bipotent TECs in relation to the emergence of mTECs and their progenitors, the control and timing of the emergence of the cTEC lineage, particularly in relation to that of mTEC progenitors, has remained elusive. In this review, we describe distinct models that explain cTEC/mTEC lineage divergence from common bipotent progenitors. In particular, we summarize recent studies in mice providing evidence that mTECs, including the auto-immune regulator^+^ subset, derive from progenitors initially endowed with phenotypic properties typically associated with the cTEC lineage. These observations support a novel “serial progression” model of TEC development, in which progenitors serially acquire cTEC lineage markers, prior to their commitment to the mTEC differentiation pathway. Gaining a better understanding of the phenotypic properties of early stages in TEC progenitor development should help in determining the mechanisms regulating cTEC/mTEC lineage development, and in strategies aimed at thymus reconstitution involving TEC therapy.

## Introduction

The thymus is dedicated to the generation of functional self-tolerant T lymphocytes, a chief effector arm of immune responses. Within thymic niches, hematopoietic progenitors, arriving from the fetal liver and bone marrow, differentiate primarily into T cells with diverse αβTCR specificities that are restricted to self-MHCs and tolerant to self-antigens (reviewed in 1,2).

The development of T cells is guided by thymic stromal cells, of which thymic epithelial cells (TECs) comprise a chief component. TECs constitute specialized structural and functional microenvironments that support critical steps of T-cell differentiation, by providing multiple cytokines, chemokines, lineage inductive ligands, and selective self-antigens that control T-cell commitment, migration, survival, proliferation, and selection (reviewed in 2,3).

## Cortical and medullary thymic epithelial niches define distinct functional microenvironments

The thymic epithelium is broadly organized into two main areas, the central medulla area in which thymic medullary epithelial cells (mTECs) reside, and the peripheral cortex area in which thymic cortical epithelial cells (cTECs) reside. These areas and the cells therein also define functionally distinct niches. cTECs have an important role during the early stages of T-cell development, driving the commitment and expansion of early T-cell progenitors via the expression of Notch ligand DLL4 4 and IL-7 5. Subsequently, cTECs mediate the selection of DP thymocytes, by expressing an array of selective self-peptides presented by MHC class I and II molecules. To accomplish this chief function as antigen-presenting cells, cTECs express a unique set of proteolytic enzymes, including a cTEC-specific proteosomal subunit β5t, a serine protease TSSP, and a lysosomal protease cathepsin L 6–8. On the other side of the “thymic yard,” mTECs play decisive roles in later stages of T-cell development, notably acting in concert with DCs to mediate the negative selection of autoreactive T cells and the generation of regulatory T cells 1–3. Crucial to the key role of mTECs in the screening of developing T cells with autoreactive TCRs is their capacity to express a myriad of tissue-restricted antigens, such as insulin 2, salivary protein 1, thyroglobulin 9. The nuclear factor auto-immune regulator (Aire) has emerged as a chief effector in tolerance induction by regulating the expression of a large array of peripheral tissue antigens (e.g. insulin 2) in a specialized subset of mTECs (reviewed in 10. Worth noting, there are other antigens associated with peripheral tissues (e.g. thyroglobulin) that are ectopically expressed in mTECs independently of Aire 11, implicating other factor(s) in the establishment of central tolerance. In addition to its key role in peripheral tissue antigen expression, Aire has recently been shown to control chemokine gene expression within the mTEC compartment 12,13.

Despite being fundamentally different in their anatomical location and functions, cTECs and mTECs share some phenotypic markers; for example, both are routinely defined by the expression of epithelial cell adhesion molecule 1/CD326 and MHC class II (MHCII) within the nonhematopoietic (CD45^−^) thymus fraction 14. However, as different analytical tools are frequently employed across studies (flow cytometry and immunohistochemical analyses), some variation exists in how researchers discriminate TEC subsets. Whereas cTECs are commonly defined by the expression of cytokeratin-8/18, CDR1, Ly51 (CD249), and ER-TR4, mTECs are distinguished by the expression of cytokeratin-5/14, MTS10, ER-TR5, and reactivity with the lectin *Ulex europaeus* agglutinin 1 14,15. The phenotypic discrimination of these cell types has considerably improved with the development of novel antibodies and the advent of newly generated reporter mice, which have allowed surveying the expression of molecules associated with cTEC- and mTEC-specific functions. Currently, cTECs are additionally identified on the basis of the expression of CD205 16, Ccrl1 17, β5t 18, and high levels of IL-7 19 and DLL4 20 expression. mTECs are usually further discriminated on the basis of the combined levels of expression of MHC class II, CD40, CD80, Aire, and most recently CCL21 16,21–24. Still, the relevance of cTEC/mTEC heterogeneity with respect to developmentally distinct stages within TEC lineages remains elusive.

In this review, we summarize recent studies in mice that have analyzed the lineage relationship between cTECs and mTECs, and we discuss possible models to explain the establishment of these two key thymic epithelial microenvironments.

## cTECs and mTECs: Same origin, but unrelated divergent lineages?

The cells in the cortical and medullary thymic epithelial compartments differentiate from bipotent thymic epithelial progenitors (TEPs) present within the embryonic 25–28 and postnatal thymus 29. The identification of TEPs, which lie at the base of the cTEC/mTEC branching point, has provided the cellular basis for a common origin of cTECs and mTECs. Despite this, the phenotypic properties and developmental requirements of bipotent TEPs are poorly understood. In mice, TEC ontogeny is initiated during early embryogenesis with the out-budding of the endoderm in the third pharyngeal pouch between day 9 and 10 of embryonic gestation (E9-E10) (reviewed in 30)^31^. At these early stages, the initiation of expression of the forkhead transcription factor Foxn1 represents a hallmark toward TEC specification 32,33. Although bipotent TEPs are maintained in Foxn1-deficient mice 29, Foxn1 is required for the initiation of a transcriptional program that engages the early differentiation of TEPs, and for the progression of cTECs and mTECs throughout distinct stages of differentiation 29,34. Recently, one study using mice with a reversible Foxn1 hypomorphic allele provided experimental evidence revealing differential requirements for Foxn1 levels in regulating these two events 34. In line with an earlier study 35, low levels of Foxn1 were shown to be sufficient to initiate the TEC differentiation program, while higher Foxn1 expression levels are needed both to achieve fully functional mature TECs and to maintain TEC lineage identity postnatally 34,35. Presently, there is no experimental evidence demonstrating that the level of Foxn1 expression modulates the determination of TEPs into the cTEC or mTEC lineages.

The discrimination between cTECs and mTECs, although particularly evident in the postnatal adult thymus, is less conspicuous at early stages of thymic organogenesis. This perhaps results from their common ancestry and the dynamic nature of TEC patterning, which is initiated during fetal development and continues throughout postnatal life 30. Yet, the precise developmental window at which cTECs and mTECs diverge, as well as the lineage relationship between TEPs and the emerging cortical and medullary progenies, remain poorly understood.

## Building epithelial microenvironments through lineage-committed progenitors

Several studies in mice have examined the development of distinct lineages downstream of bipotent TEPs. While Rodewald et al. 36 initially provided functional evidence for the existence of mTEC progenitors, advances in understanding the identity of this cell-type and stages of mTEC development have been considerably extended in the past decade. For example, successive stages of mTEC maturation have been shown to exist in mice, defined as immature MHCII^lo^CD80^lo^Aire^−^ (mTEC^lo^), mature MHCII^hi^CD80^hi^Aire^hi^ (mTEC^hi^), and recently identified terminally differentiated MHCII^lo^CD80^lo^Aire^lo^Involucrin^+^ (also residing within the originally defined mTEC^lo^) 22,37,38. Importantly, the cooperative contribution of members of the TNFR superfamily, including receptor activator of NF-κB (RANK), lymphotoxin β receptor by mTECs, is critical to the complete maturation of the Aire^+^ mTEC subset (reviewed in detail in 2,3,39). These findings led to the idea that mTECs undergo a linear differentiation process, from mTEC progenitors down to terminally differentiated mTECs, recently defined by a final post-Aire stage of maturation that is controlled by expression of Aire itself 37,40. Interestingly, studies have shown a role for lymphotoxin β receptor signaling during late stages of mTEC development, which acts to control CCL21 within the mTEC^lo^ compartment 23,41. Thus, as well as containing progenitors for mTEC^hi^ cells 21,22, mTEC^lo^ cells express molecules of known functional importance for αβ T-cell development. In addition, the expression of the tight junction proteins claudin-3 (Cld3) and Cld4 has been shown to mark a minor subset of TECs at E13 in mice, representing an mTEC progenitor subset at this developmental stage that is able to generate Aire-expressing mTECs 42. Recently, it was reported that TECs expressing high levels of Cld4 are also detected within the Foxn1-deficient E13.5 thymic primordium 34. Worth noting, while Cld3/4-expressing “mTEC progenitor-like” cells were also previously detected at E10.5 42, it remains unclear how the Cld4^hi^ cells of the nude mouse relate to functionally identified mTEC progenitors 42. In addition, a subset of CD205^−^ TECs expressing high levels of CD40 and a panel of mTEC-associated genes has been reported to arise at E14.5 16. Together, these studies provide evidence for the existence of a transitional mTEC progenitor and for direct precursor-product relationships within the mTEC lineage. However, it remains unclear whether the transitional mTEC progenitors exist immediately downstream of common bipotent TEPs, or whether such mTEC progenitors share a more intricate relationship with the nascent cTEC lineage.

In contrast to an emerging picture of discrete stages in the mTEC lineage, events occurring in the cTEC developmental pathway are less clear. However, recent studies in mice suggested the existence of cTEC-specific progenitors identified by the expression of CD205 and β5t, both hallmarks of the cTEC lineage, which appear as early as E12.5 and are absent in nude mouse embryos 16,18. While such cells were initially considered to mark a developmental stage that lies in between bipotent TEPs and mature cTECs 16, there is currently no functional evidence regarding the phenotypic identity of cTEC progenitors.

**Figure 1:**
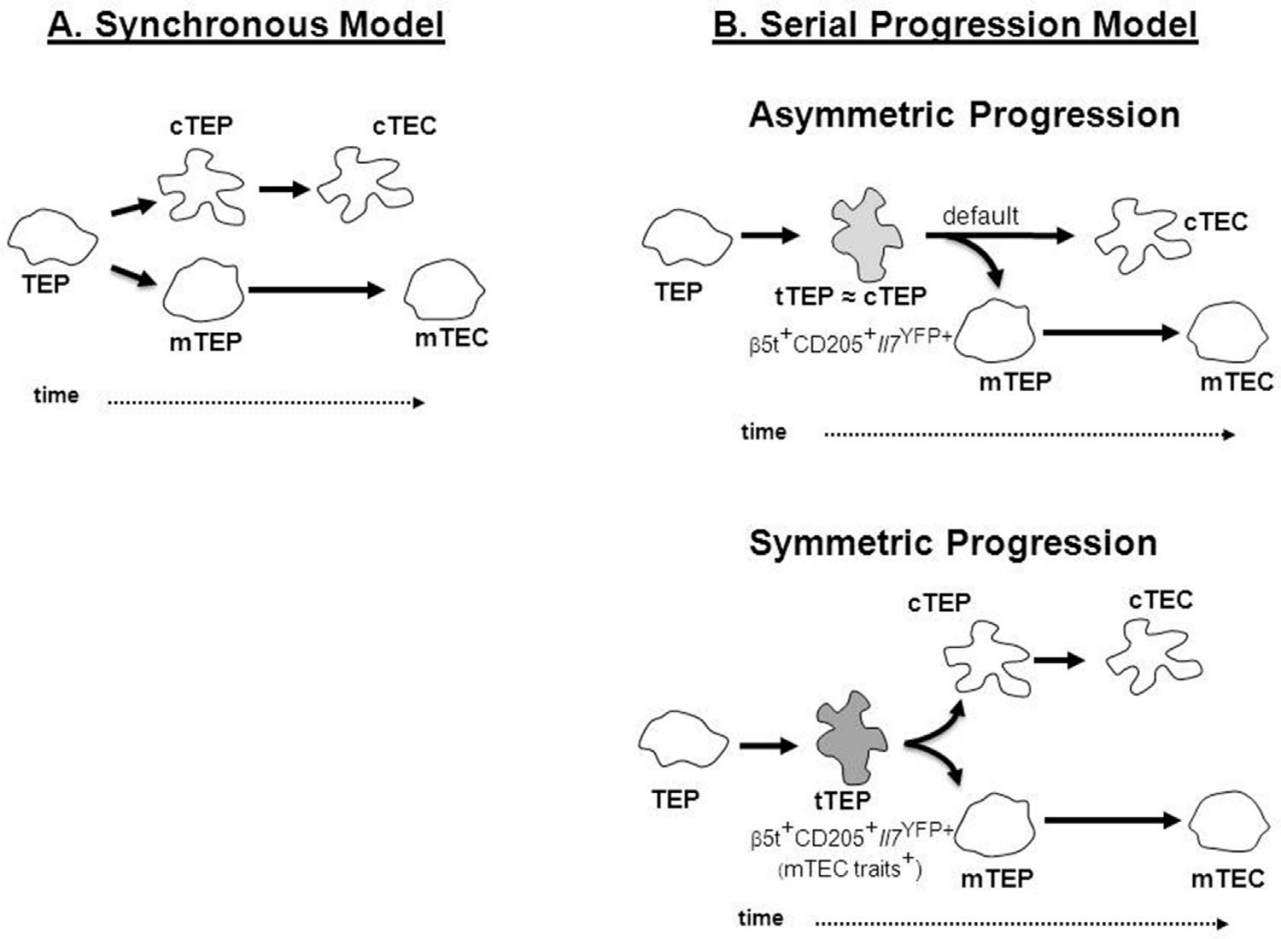
Models of thymic epithelial cell development. (A) In the “synchronous” model, uncommitted bipotent TEC progenitors (TEPs) diverge simultaneously to lineage-restricted cortical (cTEPs) and medullary (mTEPs) progenitors, which then progress into mature cTECs and mTECs. (B) In the “serial progression” model, TEPs transverse through a “transitional TEC progenitor” stage (tTEP) that expresses phenotypic and molecular traits associated with cTECs prior to the commitment into a cTEC or mTEC fate. In the asymmetric scenario (top), tTEPs are more closely linked, at the phenotypic and molecular levels, with cTEPs and have the potential to generate both mTEC progenitors and mature cTECs, with the cortical lineage being the “default” pathway. In the symmetric scenario (bottom), tTEPs express both cTEC and as-yet-unidentified mTEC traits prior to lineage specification.

Based on current findings, a simple model of TEC lineage development from bipotent TEPs can be proposed (Fig.1A), in which phenotypically and developmentally distinct cTEC progenitors and mTEC progenitors emerge in a synchronous and nonoverlapping fashion, that results in the generation of functionally distinct cTEC and mTEC compartments to control T-cell development and selection. While such a model fits well with several studies reporting the identification of lineage-restricted mTEC progenitors 16,21,42 initially identified at the clonal level 36, definitive support for this model is hampered by the lack of understanding of how and when the cTEC lineage emerges in relation to the mTEC population.

## mTECs derive from progenitors expressing cTEC markers

The identification of bipotent TEPs and evidence for the existence of compartment-restricted progenitors has cemented the concept that cTECs and mTECs follow independent differentiation pathways. Interestingly, despite several studies demonstrating the presence of mTEC progenitors 16,36,42 at early stages of embryonic thymus development, the TEC compartment at these stages has also been reported to express markers typically associated with the cTEC lineage, including CD205, β5t, and high levels of IL-7 and DLL4 16,18–20,43. Importantly, the relationship between these “cTEC marker-expressing” TECs and the mTEC lineage itself has not been directly investigated. Recently, our laboratories independently generated experimental evidence in relation to our understanding of the timing and relationship between the establishment of the cortical and medullary microenvironments, particularly with respect to the emergence of mTEC progenitors relative to the cTEC lineage 44–46.

Using a cellular approach that combines reaggregate organ cultures and ectopic thymic transplantation of phenotypically defined embryonic putative TEC progenitor populations, Baik et al. demonstrated that purified CD205^+^CD40^−^ TECs, displaying molecular traits associated with cTECs 16, comprise a source of progenitors in mice that generate both β5t/CD205-expressing cortical, and Aire-expressing medullary epithelial microenvironments in vivo 44. Ontogenetic analysis showed that, at E12.5 of gestation, functional responsiveness to the mTEC regulator RANK is evident within both CD205^+^ and CD205^−^ compartments, demonstrating that mTEC progenitors exist within the TEC subset defined by expression of the cTEC marker CD205 44. Importantly, it remains unclear whether the RANK-responsive TECs contained within the CD205^−^ subset are derived from initial CD205^+^ progeny, or whether additional RANK^+^CD205^−^ cells at early embryonic stages represent a separate stream of mTEC progenitors that do not pass through a CD205^+^ stage. Whatever the case, such observations demonstrate a “blurring” of cTEC/mTEC properties at initial stages of development in TEC populations of the embryonic thymus, and argue against the synchronous emergence of cTEC progenitors and mTEC progenitors from a common TEP pool.

In a complementary study, Ribeiro et al. 46, exploring an IL-7 reporter mouse in which YFP marks a previously identified TEC subset expressing high levels of IL-7 (*Il7*^YFP+^) 5,19, demonstrated that *Il7*^YFP+^ TECs represent a particular subset of CD205^+^Ly51^+^ cTECs throughout fetal development and perinatal life. Of note, IL-7 expression is also detected in mTECs, albeit at significantly lower levels compared to *Il7*^YFP+^ TECs 5. *Il7*^YFP+^ TECs emerge as early as E12.5 19 and comprise the majority of TECs around E13–14 of gestation 46. Employing reaggregate organ cultures (RTOCs), the authors show that E14.5 *Il7*^YFP+^ TECs can give rise to both Ly51^+^CD205^+^ cTECs and CD80^+^ mTECs 46. Thus, *Il7*^YFP+^ cells give rise to mTECs in a stepwise differentiation process via an intermediate CD80^lo^ immature mTEC stage. Still, *Il7*^YFP+^ cells do not exclusively form the entire TEC compartment at E13–14, and a smaller fraction of YFP^−^ cells is detected at this period, which steadily accumulates medullary traits as TEC maturation proceeds, including responsiveness to RANK stimulation. Similarly to the E13 CD205^−^ progenitors detected by Baik et al. 44, it remains to be determined whether YFP^−^ TECs found within the E13 thymus have a direct lineage relationship with *Il7*^YFP+^ cells or represent an alternative pathway of mTEC development. Although both studies indicate that embryonic TEC progenitors with cTEC features have the potential to generate mTECs, these studies do not determine to what degree such progenitors contribute to thymus medulla formation within the embryonic and adult thymus.

In this respect, using a knock-in mouse strategy and lineage tracing experiments, Ohigashi et al. 45 established a direct link between β5t-expressing TECs and mTECs. By crossing knock-in mice that express the recombinase Cre under the control of the endogenous β5t-encoding sequences with loxP-dependent EGFP or ZsGreen reporter mice, the authors showed that the reporter activity is not only detected in cTECs, but also in almost all mTECs, including the Aire^+^ subset, throughout ontogeny 45. β5t-Cre-mediated reporter expression was detectable even in the majority of fetal mTECs and their progenitors visualized by the high expression of K5 45 and Cld3/4 (Ohigashi and Takahama, unpublished data), indicating that all mTEC stages transverse through an early stage defined by β5t expression. As the expression of β5t is not detectable in the E11.5 thymus primordium 18,45, one can consider that β5t expression is initiated at a differentiation stage downstream of common TEPs, but prior to the branching of mTEC progenitors. The analysis of β5t fate-reporter mice corroborate that fetal and adult mTECs are almost all derived from progenitors expressing bona-fide cTEC traits under normal physiological circumstances.

Collectively, these observations demonstrate that the medullary lineage is derived from progenitors defined by a cTEC “footprint,” thereby arguing against a model of TEC development involving the synchronous emergence of distinct pools of cTEC progenitors and mTEC progenitors from bipotent TEPs. Based on these findings, we propose an alternative model of TEC development, referred to here as the “serial progression” model (Fig.1B), to explain these findings in relation to the establishment of cortical and medullary microenvironments. This model points to the existence of a novel transitional progenitor stage in the TEC differentiation pathway currently defined by the expression of a set of cTEC-associated genes, including β5t, CD205, and high levels of IL-7. As the development of Aire-expressing mTECs is not affected in β5t- 6,47, CD205- 48, or IL-7-deficient mice (Rodrigues and Alves, unpublished data), these findings suggest that the expression of these cTEC traits solely identifies transitional TEC developmental stages, rather than being directly implicated in the divergence of the mTEC lineage. While such cortical attributes may be transiently transcribed at an early phase of both cTEC and mTEC lineages, and then either enhanced during the default cTEC development or progressively lost during differentiation into mature mTECs, this model supports a process in which cTEC and mTEC lineages follow asymmetrical differentiation pathways from bipotent TEPs. In this differentiation route, transitional and cortical TEC progenitors are more closely related at phenotypic, molecular, and functional levels (Fig.1B). However, one cannot exclude that TEPs progress through the transitional TEP state prior to the commitment into the cTEC and mTEC lineages. In this variant “symmetric serial progression” model (Fig.1B), one considers that transitional TEPs may begin coexpressing cTEC- in addition to, yet unresolved mTEC-associated genes. Once the lineage fate is programmed into cTEPs or mTEPs, the expression of lineage-specific molecules is permitted and the expression of molecules specific for another lineage is terminated. Further studies on the molecular mechanisms regulating the dynamics of the serial progression of the cTEC and mTEC lineages are warranted.

## Conclusions

The recent developments discussed in this review provide an important change in the understanding of TEC lineage specification. The identification of transitional progenitors with cortical traits indicates that cTECs and mTECs might share a more intricate lineage relationship downstream of bipotent TEPs than previously recognized. This raises more general questions of how TEC differentiation is balanced to create functionally diverse epithelial microenvironments. Given the considerable plasticity in the lineage potential of transitional TEPs, it will be important to determine whether these cells exist in the adult thymus. Additionally, we still lack evidence on how transcriptional and epigenetic changes contribute to the specification of TEC lineages.

The “asymmetrical serial progression” model implies that the specification into the cortical lineage is the default pathway downstream of the TEC bipotent progenitors. From an evolutionary point of view, one can envisage that TECs from ancient vertebrates initially evolved with the functional capacity to commit thymic seeding precursors into the T-cell lineage and to restrict the immense diversity of generated TCR specificities to self-MHC molecules. In this scenario, the emergence of the mTEC lineage is a sophisticated event that evolved later to guarantee self-tolerance, either through the purging of autoreactive T cells or the generation of T regulatory cells 49. It is interesting to note that distinct lymphopoietic microenvironments are also detected in the recently identified thymoids of jawless vertebrates 50. Further studies on the TEC compartment of primitive vertebrates should elucidate whether these niches represent functional equivalents to the cortical and medullary lineages found in the thymus of jawed vertebrates. Knowledge in this area will enable us to gain a more complete appreciation of the fundamental rules that govern the complex diversification of TECs in mammals.

Interestingly, the concept presented here seems to extend to other cell types and tissues, in which the indistinctness between progenitors and lineage-specific phenotypes is observed. For example, during the development of the central nervous system, both glial and neuronal cell lineages emerge from a common progenitor that expresses proteolipid protein, a predominant myelin neuronal-restricted component 51.

Taken together, the recent studies discussed here provide a clearer definition of TEC progenitors capable of giving rise to both cTECs and mTECs. This could have important implications in relation to cellular immunotherapy approaches involving thymus regeneration/replacement 52, in which functional and self-tolerant T-cell production depends upon the presence of both thymic compartments.

## Abbreviations

Aire: auto-immune regulator
Cld: claudin
cTEC: cortical TEC
mTEC: medullary TEC
RANK: receptor activator of NF-κB
TEC: thymic epithelial cell
TEP: thymic epithelial progenitor
